# Dynamic Speciation as a Hidden Driver of Reactivity: Lessons from Organozinc Chemistry

**DOI:** 10.1021/acs.accounts.6c00598

**Published:** 2026-10-01

**Authors:** Michał Terlecki, Iwona Justyniak, Krzysztof Budny-Godlewski, Janusz Lewiński

**Affiliations:** † Faculty of Chemistry, 49566 Warsaw University of Technology, Noakowskiego 3, 00-664 Warsaw, Poland; ‡ Institute of Physical Chemistry, Polish Academy of Sciences, Kasprzaka 44/52, 01-224 Warsaw, Poland

## Abstract

Organometallic reactivity is traditionally rationalized in terms of well-defined molecular structures. In this Account, we argue that for organozinc compounds, this picture is incomplete: their reactivity is governed not by isolated molecules but by dynamic speciation landscapes—equilibrating Lewis acid–base adducts, interconverting aggregates, species generated by Schlenk redistribution, transient intermediates, and phase-confined assemblies whose composition evolves throughout a reaction. Drawing on more than two decades of our group’s work, we show that reaction outcomes emerge from the identity and evolution of these reactive ensembles rather than from any single species that can be crystallized and assigned a formula. Moving from static structures to reactive landscapes, we first consider molecular complexes as programmable reactive platforms, in which aggregation and donor coordination generate families of interconverting species that can be deliberately tuned. We then recast Schlenk equilibria, long regarded as a passive redistribution nuisance, as an active determinant of reactivity: by continuously reshaping the pool of available reagents, they define which species are present when the reaction occurs and thereby redirect its course.

Asymmetric catalysis puts the ensemble picture to its sharpest test. For example, chiral amplification in the dialkylzinc addition to aldehydes is classically explained by an equilibrium between catalytically active and dormant aggregates, a two-level picture of the reactive ensemble. Our work on bifunctional alcohols indicates that this ensemble is richer: we isolated an unprecedented tetranuclear zinc aggregate, pointing to higher-nuclearity species as possible sources of the catalytically competent centers. The unexpected incorporation of the solvent into one such species further showed that even the reaction medium participates in shaping the ensemble. Conversely, where the ensemble proved simple enough to define—as in the epoxidation of enones by zinc alkylperoxides—it became a design tool: systematic ligand modification delivered enantioselectivities reaching 91% *ee*. Speciation control overturns an even longer-standing assumption. The reaction of organozinc compounds with O_2_, long treated as an uncontrolled radical process, emerges instead as a sequence of elementary steps governed by latent Lewis acidity, inner-sphere electron transfer (ISET), and product inhibition—steps that allow reactive intermediates to be intercepted, giving controlled access to alkylzinc alkylperoxides and related oxygenated species.

Beyond homogeneous solution, we show how phase-dependent speciation modulates reactivity in the solid state: mechanochemical and phase-confined environments, together with supramolecular self-assembly, stabilize ensembles and open pathways—and give access to functional materials—inaccessible in solution, establishing a continuum linking molecular speciation to materials formation.

Across these systems, one principle recurs: the reactive species is often not an isolated molecule but a transient member of an evolving ensemble. We close with three challenges we consider decisive: dynamic speciation must be monitored directly under operating conditions, combining in situ spectroscopy, diffraction, computation, and kinetics; aggregation, donor coordination, Schlenk equilibria, and confinement should be treated as design variables rather than complications; and mastering organozinc speciation offers a route to more predictable reactivity and to rational molecular-to-material transformations.

## Graphical abstract

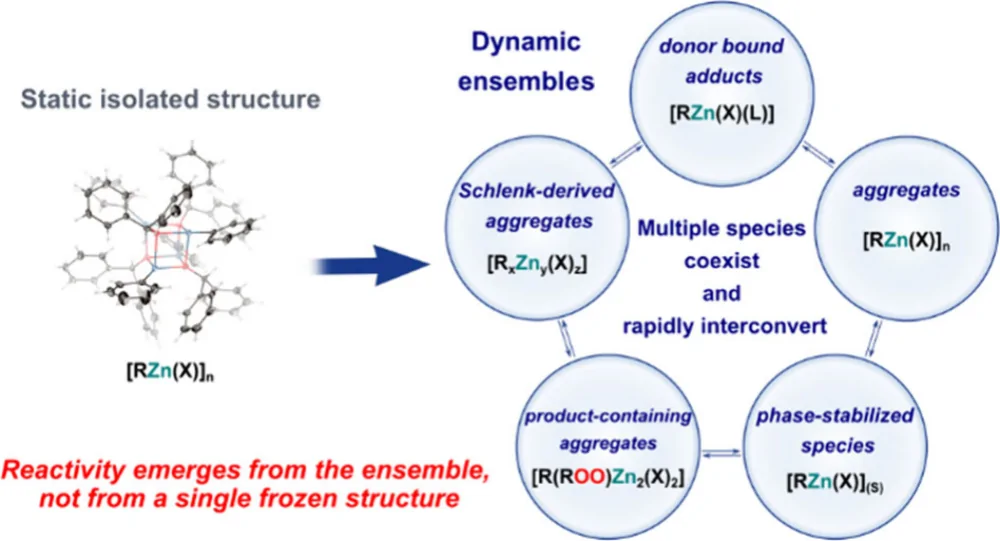

## Key References

-

Lewiński, J. ; Marciniak, W. ; Lipkowski, J. ; Justyniak, I. New Insights into the Reaction of Zinc Alkyls with Dioxygen. J. Am. Chem. Soc. 2003, 125 (42), 12698–12699 10.1021/ja036020t14558804.^1^ This study established that organozinc oxygenation proceeds through structurally diverse, aggregation-dependent pathways rather than a single uniform mechanism. It provides an early foundation for this Account’s central argument that reactive speciation governs chemical outcome.
-

Budny-Godlewski, K. ; Piekarski, D. G. ; Justyniak, I. ; Leszczyński, M. K. ; Nawrocki, J. ; Kubas, A. ; Lewiński, J. Uncovering Factors Controlling Reactivity of Metal-TEMPO Reaction Systems in the Solid State and Solution. Chem. Eur. J. 2024, 30 (54), e202401968 10.1002/chem.20240196838801170.^2^ This paper shows how subtle changes in metal identity, coordination environment, and reaction phase redirect metal–aminoxyl reactivity. It illustrates the broader principle that observed reactivity emerges from a dynamic interplay of structure, speciation, and medium.
-

Sokołowski, K. ; Justyniak, I. ; Terlecki, M. ; Fairen-Jimenez, D. ; Bury, W. ; Budny-Godlewski, K. ; Nawrocki, J. ; Kościelski, M. ; Lewiński, J. Stabilization toward Air and Structure Determination of Pyrophoric ZnR_2_ Compounds via Supramolecular Encapsulation. Sci. Adv. 2025, 11 (17), eadt7372 10.1126/sciadv.adt7372PMC1202463540279417.^3^ This work demonstrates that highly reactive organozinc species can be trapped through supramolecular confinement, isolated, and structurally characterized. It underscores the distinction between capturing a single molecular state and understanding the ensemble responsible for reactivity under unconstrained conditions.
-

Terlecki, M. ; Zelga, K. ; Justyniak, I. ; Bernatowicz, P. ; Pietrzak, T. ; Leszczyński, M. K. ; Lewiński, J. Unmasking the Role of Schlenk Equilibria in Governing the Oxygenation of Heteroleptic Alkylzinc Complexes. Chem. Eur. J. 2026, 32 (32), e71129 10.1002/chem.71129PMC1350567242139697.^4^ This paper directly demonstrates that Schlenk equilibria determine which organozinc species reacts with dioxygen and which products are ultimately formed. It provides the clearest experimental validation of this Account’s reactive-ensemble paradigm.

## 1 Introduction

For more than 160 years, organometallic chemistry has advanced through the characterization of well-defined molecular species. From Frankland’s pioneering organozinc compounds^5,6^ to the modern reagents that underpin asymmetric synthesis, catalytic polymerization, and materials science, mechanistic interpretation has largely relied on the assumption that an isolated molecular structure adequately represents the chemically relevant species.^7–10^ Yet many organometallic systems exist as dynamic ensembles of interconverting species generated through aggregation, Schlenk-type equilibria, ligand redistribution, solvation, and product-induced structural reorganization.^1,4,11–14^ Consequently, the isolated structure may differ fundamentally from the ensemble governing reactivity. Organozinc chemistry provides an exceptional platform for exploring this concept (Figure 1). The relatively labile Zn–C bond, flexible coordination environment, and strong tendency toward aggregation generate rich speciation landscapes. Their sensitivity to ligand and alkyl identity, concentration, solvent, interactions with products of their transformations, and reaction phase makes organozinc systems ideal for probing how evolving speciation governs reactivity across length scales.

**Figure 1:**
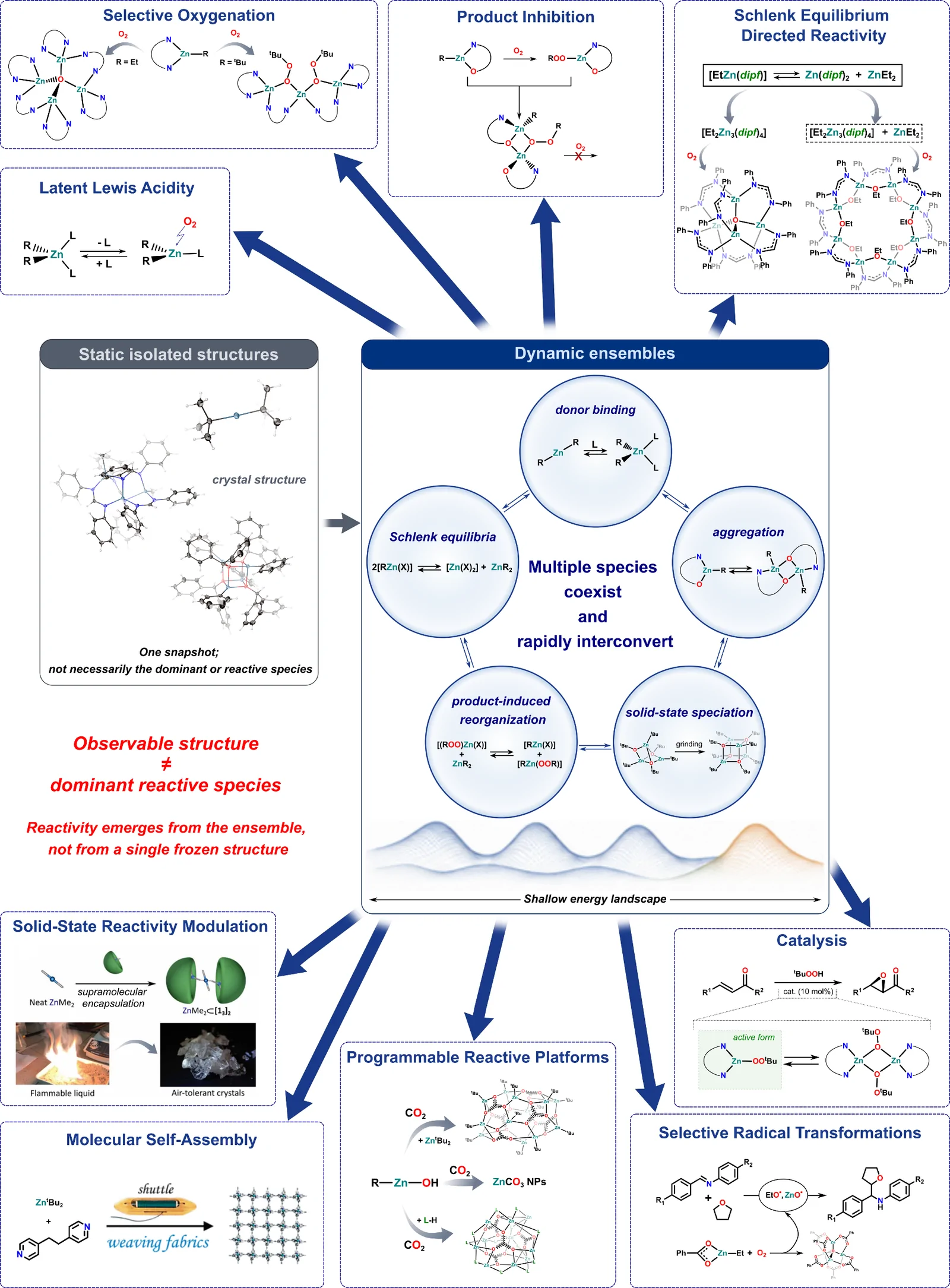
Schematic representation of the conceptual shift from static molecular structures to dynamic reactive ensembles and its implications for organozinc chemistry.

The limitations of oversimplified speciation models are evident across major areas of organozinc chemistry. In asymmetric ZnR_2_ addition to aldehydes, including the Soai autocatalytic reaction, the identities of the catalytically relevant zinc alkoxide aggregates remain under debate.^15,16^ Likewise, the prevailing model of organozinc-initiated radical chemistry assigned initiation in ZnR_2_/O_2_ systems to carbon-centered radicals,^17^ whereas our mechanistic studies identified oxygen-centered radicals as the initiating species.^18^ Together, these examples show that reliable mechanistic interpretation requires consideration of the full dynamic speciation landscape rather than isolated molecular structures.

Drawing on more than two decades of our systematic studies, this Account develops an ensemble-based framework for organozinc reactivity (Figure 1), with particular emphasis on Schlenk equilibria, dynamic structural reorganization, latent Lewis acidity, and product inhibition. We examine their consequences for asymmetric catalysis and organozinc oxygenation and finally extend this perspective to phase-dependent and supramolecular reactivity.

## 2 From Static Structures to Reactive Landscapes: Molecular Complex Platform, Schlenk Equilibria, Chiral Amplification, Product Inhibition, and Latent Lewis Acidity

### 2.1 Molecular Complexes as Programmable Reactive Platforms

Although homoleptic ZnR_2_ alkylzinc compounds are among the oldest known organometallic species,^5^ their structural characterization remained elusive for a long time. One of the earliest crystallographic studies, reported in 1990, revealed that diphenylzinc forms weakly associated dimers, [ZnPh_2_]_2_.^19^ Most ZnR_2_ compounds, however, adopt monomeric structures in both the solid state and in noncoordinating solvents, featuring a linear coordination geometry at zinc.^20,21^ In 2007, we reported the crystal structure of Zn^t^Bu_2_,^22^ which contrasts with the previously characterized dimeric magnesium analogue Mg^t^Bu_2_ ^23^ and highlights the different propensities of these metals to form alkyl-bridged aggregates (Figure 2a). Structural characterization of the lighter homologues ZnMe_2_ and ZnEt_2_ proved more challenging due to their volatile and pyrophoric nature and was achieved only in 2011.^21^ More recently, we also demonstrated the structural characterization of these molecules within a novel crystalline environment provided by supramolecular capsules.^3^

**Figure 2:**
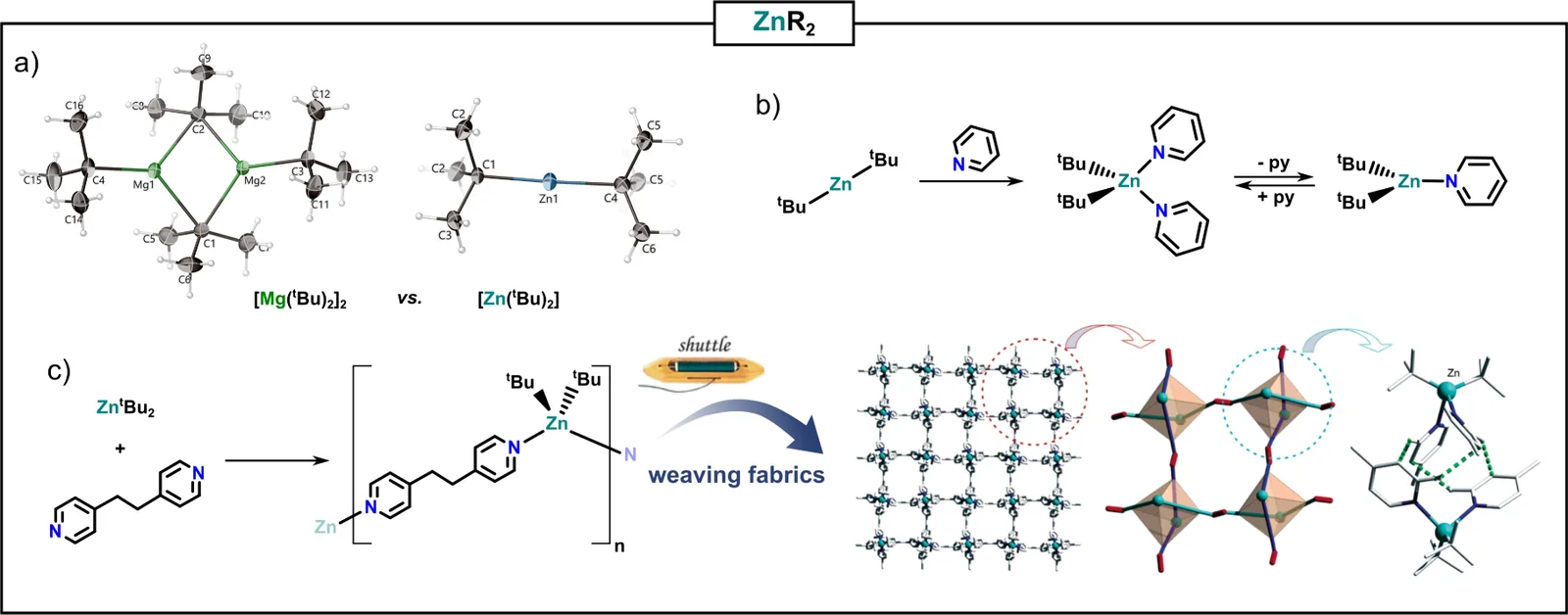
(a) Comparison of the crystal structures of di-*tert*-butylmagnesium and di-*tert*-butylzinc. (b) Dynamic equilibrium in the Zn^t^Bu_2_/pyridine system. (c) Construction of 2D supramolecular fabrics from Zn^t^Bu_2_ nodes and *bpe* linkers. Adapted with permission from ref 22. Copyright 2007 American Chemical Society.

Beyond their apparent structural simplicity, ZnR_2_ compounds are highly responsive to their chemical environment. Their Lewis acidity enables reversible coordination of neutral donors, generating coordination equilibria that strongly depend on donor identity, solvent, and concentration. Such interactions increase the coordination number at zinc and can profoundly alter molecular and supramolecular organization (Figure 2b).^22,24–27^ An especially illustrative example is provided by our use of ZnR_2_ compounds as nodes in coordination supramolecular frameworks. Combining Zn^t^Bu_2_ with the flexible linker 1,2-bis­(4-pyridyl)­ethane (*bpe*) afforded the coordination polymer [Zn­(^t^Bu)_2_(*bpe*)]_n_, featuring an organometallic two-dimensional supramolecular “fabric” architecture (Figure 2c).^22^ Its woven topology arises from Zn–N coordination reinforced by cooperative C–H···π interactions. Interestingly, the corresponding adduct with the more rigid 1,2-bis­(4-pyridyl)­ethylene linker does not exhibit weaving, highlighting the crucial role of linker conformational flexibility and secondary interactions.

A complementary source of structural diversity arises from the polarized, Brønsted-basic Zn–C bond. Protonolysis of ZnR_2_ with suitable L-H proligands readily generates heteroleptic [RZn­(L)] derivatives whose molecular organization is strongly influenced by the supporting ligand, alkyl group and coordinating medium. Replacing one alkyl group with an anionic ligand substantially reshapes the coordination preferences and Lewis acidity of the zinc center, opening additional pathways for donor coordination and aggregation. Consequently, heteroleptic [RZn­(L)] complexes display considerable structural versatility that can be systematically tuned through the steric and electronic properties of the supporting ligand, encompassing alkoxides, carboxylates, pyrrolyls, amidates, amidinates, guanidinates, and related systems (*vide infra*). Notably, their solution-state behavior is often more complex than suggested by single-crystal structures. Even well-defined heteroleptic [RZn­(L)] complexes participate in dynamic equilibria involving aggregation, ligand redistribution, and donor coordination, further modulated by solvent, concentration, and reaction products. Thus, solid-state species need not represent the reactive forms present in solution. Reliable structure–reactivity relationships therefore require consideration of the underlying speciation landscape rather than crystallographic structures alone.

A particularly revealing case is provided by the organozinc alkoxides [RZn­(OR′)]. For less hindered derivatives, the dominant preferred structural motif is the tetrameric heterocubane [RZn­(OR′)]_4_,^10,28–31^ whereas sterically demanding substituents may stabilize dimeric [RZn­(OR′)]_2_ structures (Figure 3a).^32,33^ Our studies showed, however, that this classical dimer–cubane picture captures only part of the accessible speciation landscape. Controlled solid-state desolvation of the THF-solvated dimer [^t^BuZn­(O^t^Bu)­(THF)]_2_ under mild vacuum afforded the unique metastable trimer [^t^BuZn­(O^t^Bu)]_3_, a connectivity pattern with no precedent in simple organozinc alkoxide chemistry (Figure 3b).^34^ This trimer cannot be accessed by direct crystallization from solution, where the thermodynamic heterocubane [^t^BuZn­(O^t^Bu)]_4_ forms instead. Remarkably, mechanical grinding converts the trimer into the cubane within minutes, whereas the analogous transformation in solution requires several hours. The odd-to-even change in RZnOR units excludes simple intracluster relaxation and instead requires Zn–O bond cleavage, reassembly, and redistribution. Thus, mechanochemical activation does not merely accelerate solution chemistry, but opens a distinct solid-state speciation pathway.

**Figure 3:**
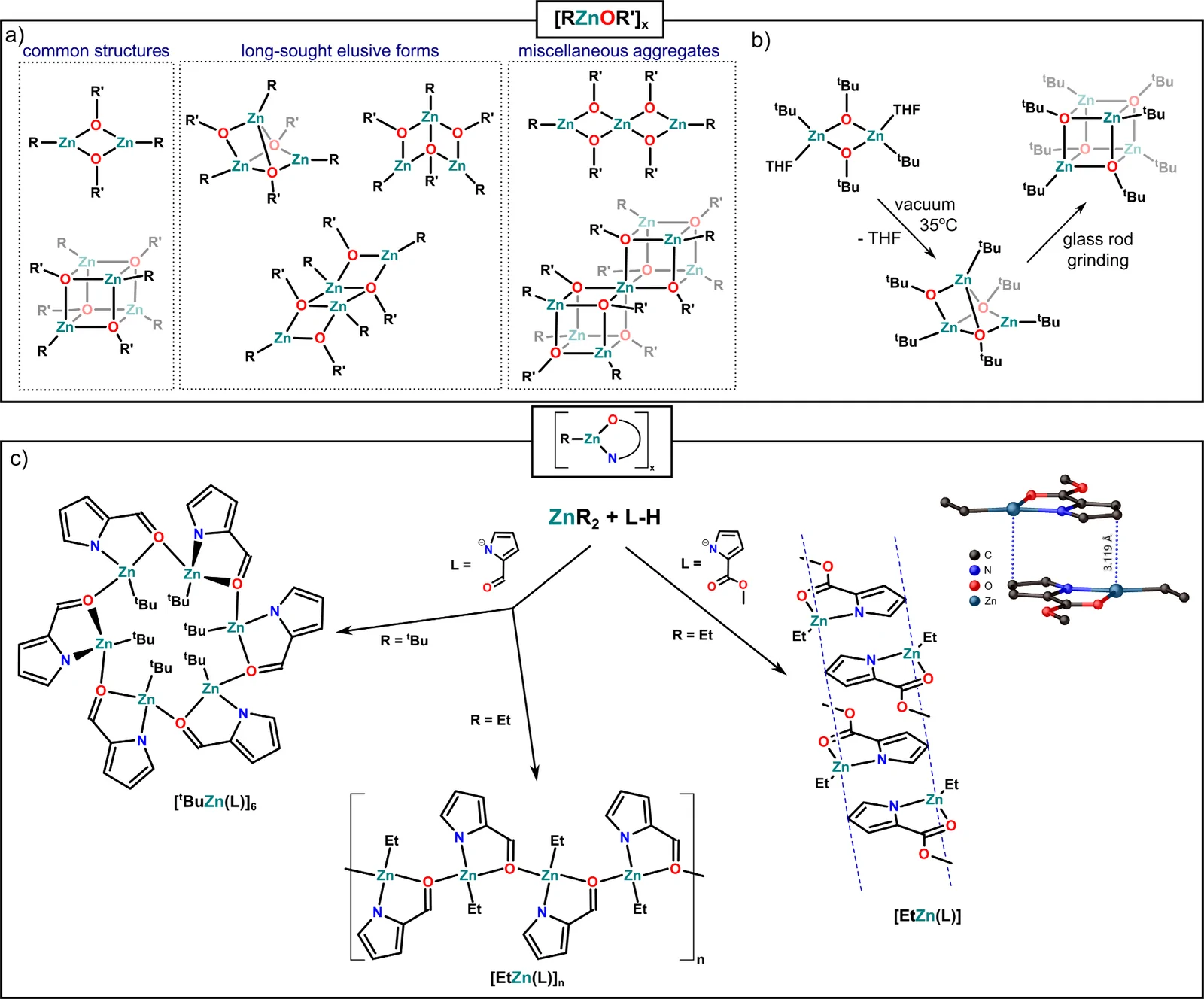
(a) Structural diversity and dynamic speciation of alkylzinc complexes supported by simple alkoxide ligands. (b) Solid-state transformations in [^t^BuZn­(O^t^Bu)]_n_ system. (c) Impact of subtle modifications of the O,N-bidentate ligand framework or the Zn–R group on the structural organization of [RZn­{O,N}] complexes.

This phase-dependent access to a metastable cluster anticipated the broader conclusion that crystallographic structures of organozinc alkoxides are snapshots of dynamic speciation landscapes rather than fixed molecular identities. More recent studies identified additional roof-like and ladder-type motifs,^35^ while rare Schlenk-redistributed aggregates, such as heptanuclear corner-shared double-cubane [(RZn)_6_Zn­(OR′)_8_] ^1,28,36,37^ and spiro-type trinuclear derivatives [(RZn)_2_Zn­(OR′)_4_],^35,38,39^ illustrate departures from simple [RZnOR′]_n_ stoichiometry. Complementary multinuclear NMR studies showed that these compounds exist in solution as dynamic ensembles shaped by aggregation–disaggregation and ZnR_2_-mediated Schlenk-type ligand redistribution.^35^ Thus, even the simple formula [RZn­(OR′)] masks a network of interconverting species whose composition depends on sterics, donor binding, concentration, solvent, crystallization history, and phase.

Similar structural versatility and solution dynamics are observed for alkylzinc complexes supported by O,O′-,^40^ O,N-,^41–43^ and N,N′-bidentate^44,45^ ligands. Alkylzinc aminoalcoholates frequently crystallize as dimers, whereas solution studies reveal equilibria involving monomeric and higher aggregates.^41,42^ A particularly illustrative case is provided by alkylzinc complexes supported by bifunctional pyrrolide ligands (Figure 3c). The ethylzinc complex of methyl 1H-pyrrole-2-carboxylate crystallizes as discrete three-coordinate [EtZn­{O,N}] species, in which low-coordinate Zn­(II) centers are stabilized by intermolecular Zn···π interactions one-dimensional supramolecular chains.^43^ Replacing the ester group with an aldehyde functionality affords a one-dimensional coordination polymer [EtZn­{O,N}]_n_ with coordinatively saturated zinc centers, whereas substitution of the ethyl group with *tert*-butyl redirects the assembly toward discrete hexameric aggregates (Figure 3c). Together, these examples illustrate how subtle modifications of either the ligand framework or the Zn-bound alkyl group profoundly alter the speciation and structural organization of alkylzinc complexes.

The pronounced structural flexibility of organozinc complexes makes them ideal molecular platforms for programming chemical reactivity, enabling the rational design of reactive systems in which the composition and speciation of the reactive ensemble dictate the course of chemical transformations. Representative examples are provided by our studies on the reactivity of alkylzinc compounds toward small inorganic molecules. For instance, we developed efficient synthetic routes to the well-defined zinc-oxo [Zn_4_(μ_4_-O)­(O_2_CPh)_6_] and zinc-sulfido [Zn_3_(μ_3_-S)­(O_2_CPh)_4_(THF)]_2_ clusters through the reactions of hexameric ethylzinc benzoate with O_2_ and S_8_, respectively (Figure 4).^46^ These transformations demonstrated how a preorganized alkylzinc carboxylate scaffold can direct the assembly of structurally distinct heteroatom-centered clusters. In subsequent studies, we established a complementary strategy for the synthesis of [Zn_4_(μ_4_-O)­(O_2_CR)_6_] zinc oxo clusters through hydrolysis of predesigned alkylzinc precursors.^47^ Matching the precursor and target cluster stoichiometry allowed for atom-economical and high-yield syntheses under mild conditions. Related organozinc hydroxide chemistry, reviewed elsewhere, likewise shows that the Zn-bound alkyl group and supporting ligand govern stability and aggregation.^48^ A parallel example outside strict organozinc chemistry is the oxozinc cluster [Zn_4_(μ_4_-O)­(O_2_CCF_3_)_6_], whose enzyme-like O-selective acylation illustrates how a defined multinuclear zinc architecture can support unusual chemoselectivity.^49^

**Figure 4:**
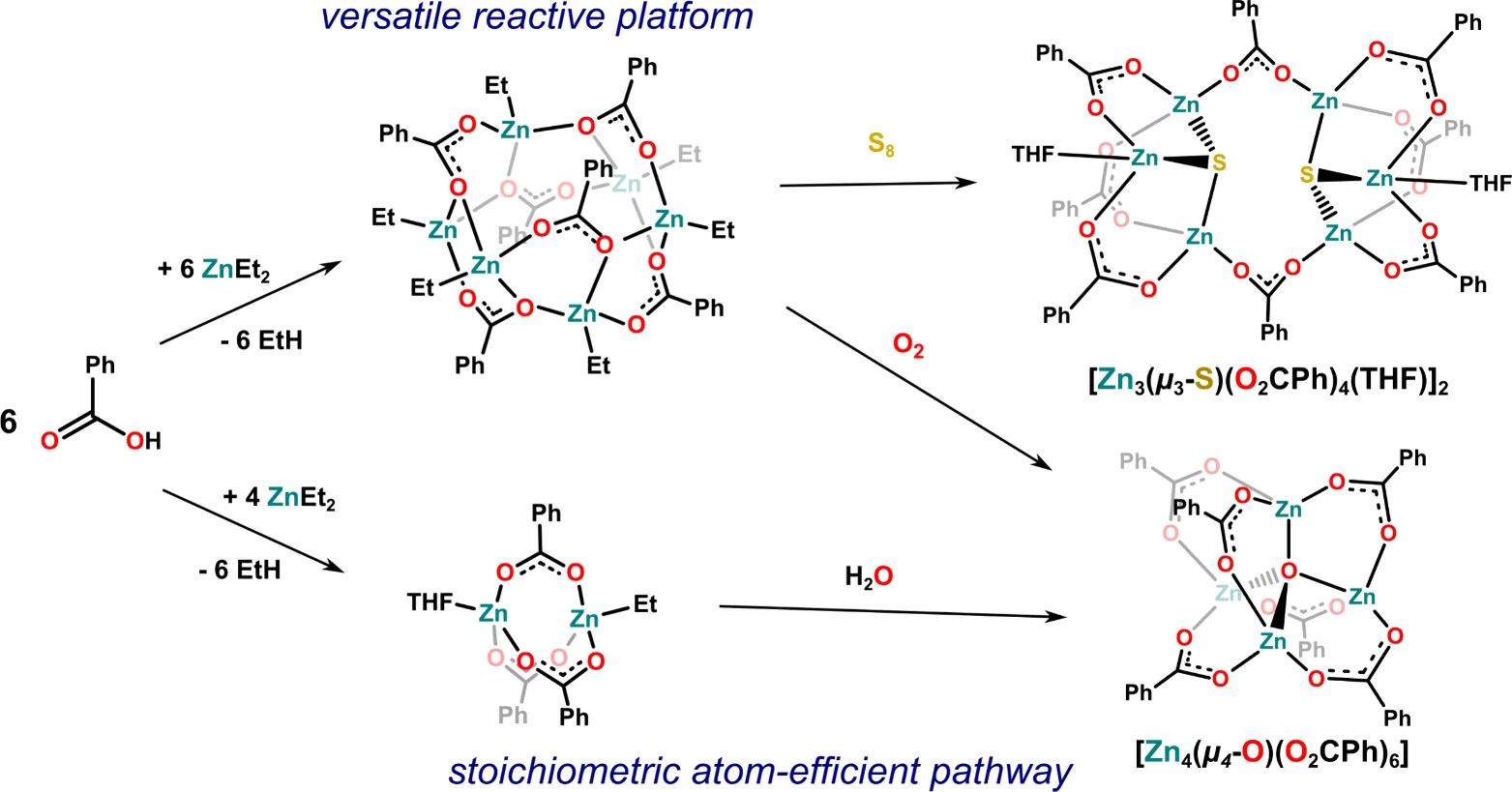
Alkylzinc benzoates as precursors of zinc oxo and sulfido clusters.

The same rationale guided the synthesis of zinc carbonate clusters from bifunctional alkylzinc hydroxide precursors. Because RZnOH species contain mutually reactive Zn–C and Zn–OH functionalities, they are generally unstable and readily decompose into alkylzinc (oxo)­hydroxides (Figure 5a).^48,50^ A major advance was the isolation of [^t^BuZnOH]_6_, which adopts a well-defined drum-like hexameric structure.^51^ Its reactions with CO_2_ revealed a pronounced ensemble effect: in the absence of additives, carbonation leads to mesoporous ZnCO_3_ nanoparticles, whereas excess Zn^t^Bu_2_ redirects the system toward the molecular cluster [(^t^BuZn)_2_CO_3_]_6_ (Figure 5c).^52,53^ Combined experimental and computational studies identified donor-stabilized [^t^BuZn­(OH)­(THF)]_2_ dimers, as the key reactive species, while proton transfer from bicarbonate intermediates governs carbonate-cluster formation (Figure 5b). A complementary example is provided by the preorganized tetranuclear alkylzinc hydroxide complex [^t^Bu_2_Zn_4_(OH)_2_(L)_4_], supported by chelating 8-quinolinolate ligands (L).^54^ Carbonation of this precursor afforded the discrete nanosized cluster [(LZn)_6_(ZnCO_3_)_4_], which subsequently self-assemble into a 3D microporous diamondoid network **WUT-1­(Zn)** with high surface area and substantial uptake of N_2_, H_2_, CO_2_, and CH_4_ (Figure 5d).^55^ The same architectural principle extends beyond zinc: an analogous decanuclear nickel­(II) carbonato cluster forms two interchangeable porous frameworks, diamondoid **WUT-1­(Ni)** and pyrite **WUT-2­(Ni)**, both exhibiting excellent gas adsorption selectivity.^56^ Collectively, these systems show how precursor organization and ensemble composition direct CO_2_ fixation across molecular, supramolecular, and materials regimes.

**Figure 5:**
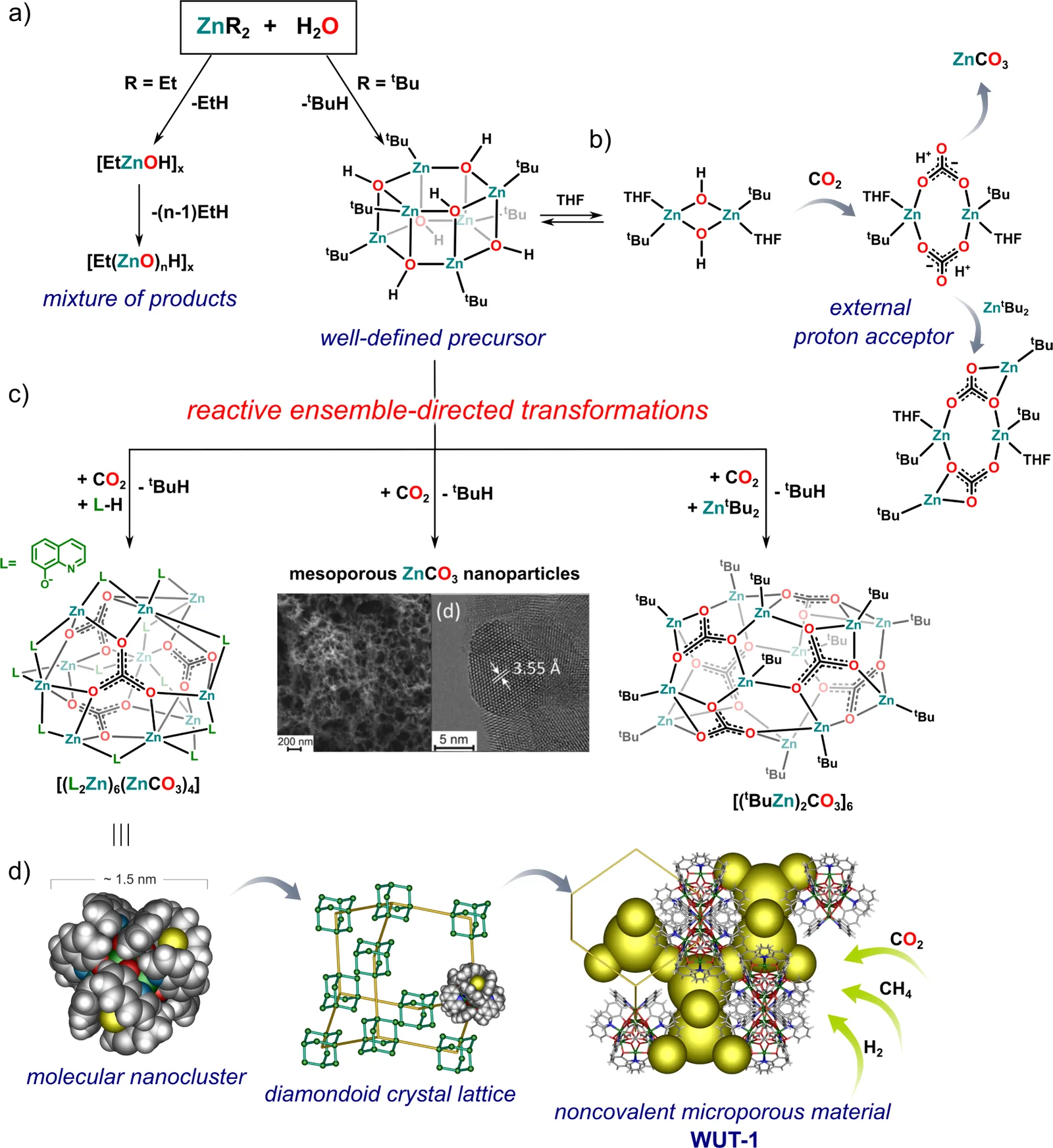
Alkylzinc hydroxides as precursors to molecular and bulk zinc carbonates: (a) influence of the Zn–R group on RZnOH species stability; (b) reaction pathways to various zinc carbonate products; (c) ensemble-directed transformations of [^t^BuZnOH]_6_ with CO_2_; (d) self-assembly of [(LZn)_6_(ZnCO_3_)_4_] into the microporous **WUT-1** framework. SEM/TEM images adapted with permission from ref 52. Copyright 2013 Royal Society of Chemistry.

What emerges is the central theme of this Account: the chemistry of organozinc systems is determined not by the structure of an isolated molecular complex but by the reactive landscape arising from the interplay between structural flexibility and dynamic speciation. The following sections examine the principal mechanisms reshaping this reactive landscape, beginning with Schlenk equilibria as an underappreciated determinant of solution-state speciation.

### 2.2 Schlenk Equilibria: An Underappreciated Determinant of Organozinc Reactivity

The solution-state composition of heteroleptic organometallic systems is often governed by Schlenk equilibria, yet their mechanistic consequences remain underappreciated. Rather than establishing a single dominant molecular species, these equilibria generate dynamic ensembles of continuously interconverting organometallic compounds (Figure 6a). First recognized nearly a century ago in the context of Grignard reagents,^57^ Schlenk equilibria have since been documented in systems beyond magnesium, including organozinc,^12,27,45,58–60^ alkaline-earth,^61,62^ and rare-earth compounds.^63^ Nevertheless, they are still largely regarded as a source of structural diversity rather than as active determinants of reaction pathways and chemical reactivity. In alkylzinc chemistry, Schlenk equilibria are generally shifted toward heteroleptic [RZn­(L)] species, which are frequently isolated as well-defined [RZn­(L)]_n_ aggregates (Figure 6b) and have long been regarded as the sole chemically relevant species. However, our systematic studies showed that several ligand classes, including alkoxides,^35^ pyrazolates,^59,60^ guanidinates,^64^ and amidinates,^45^ promote extensive Schlenk equilibrium-mediated ligand scrambling, generating redistributed assemblies from coexisting [RZn­(L)] and [Zn­(L)_2_] species. These clusters, with stoichiometries such as [R_2_Zn_3_(L)_4_], [R_2_Zn_4_(L)_6_], and [R_3_Zn_4_(L)_5_], were long regarded as structural curiosities. Yet, their existence demonstrates that Schlenk equilibria actively shape alkylzinc speciation, generating reactive ensembles that cannot be adequately represented by conventional aggregates with an equimolar R:L ratio alone.

**Figure 6:**
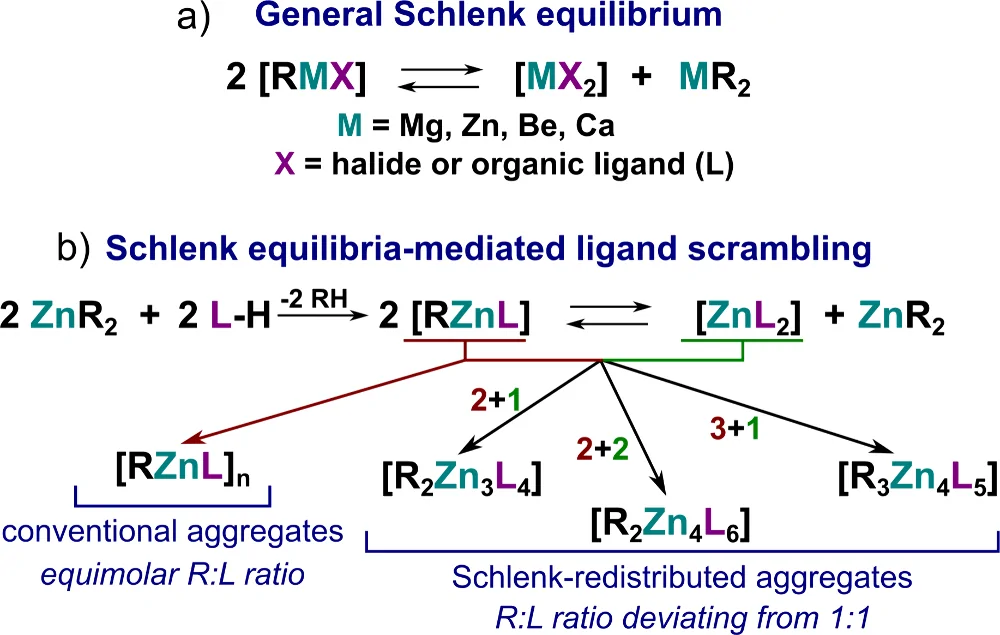
Schematic representation of (a) general Schlenk equilibrium in heteroleptic organometallic compounds and (b) Schlenk equilibria-mediated ligand scrambling in heteroleptic alkylzinc complexes.

Our studies on alkylzinc amidinates demonstrate how subtle changes in ligand structure can fundamentally reshape Schlenk equilibria and, consequently, the reactive ensemble.^45^ Derivatives of benzamidine (*bza*-H) and N,N′-diphenylformamidine (*dipf*-H) rather than persisting as simple [RZn­(L)]_n_ species, exhibit a pronounced tendency to undergo Schlenk-type ligand redistribution, generating thermodynamically preferred aggregates with R:L ratios deviating from 1:1 (Figure 7a). The synthesis output was sensitive to the amidinate N-substituents: N,N′-unsubstituted *bza* affords complex mixtures of redistributed species ([R_3_Zn_4_(*bza*)_5_], [R_4_Zn_5_(*bza*)_6_], [RZn_3_(*bza*)_5_], among others), whereas N,N′-diaromatic-substituted *dipf* converges to the exceptionally stable [R_2_Zn_3_(*dipf*)_4_] aggregate, reinforced by intramolecular CH···π and π···π interactions in the secondary coordination sphere. In both cases, the Schlenk-redistributed complexes coexist with free ZnR_2_, reflecting the underlying equilibria.

**Figure 7:**
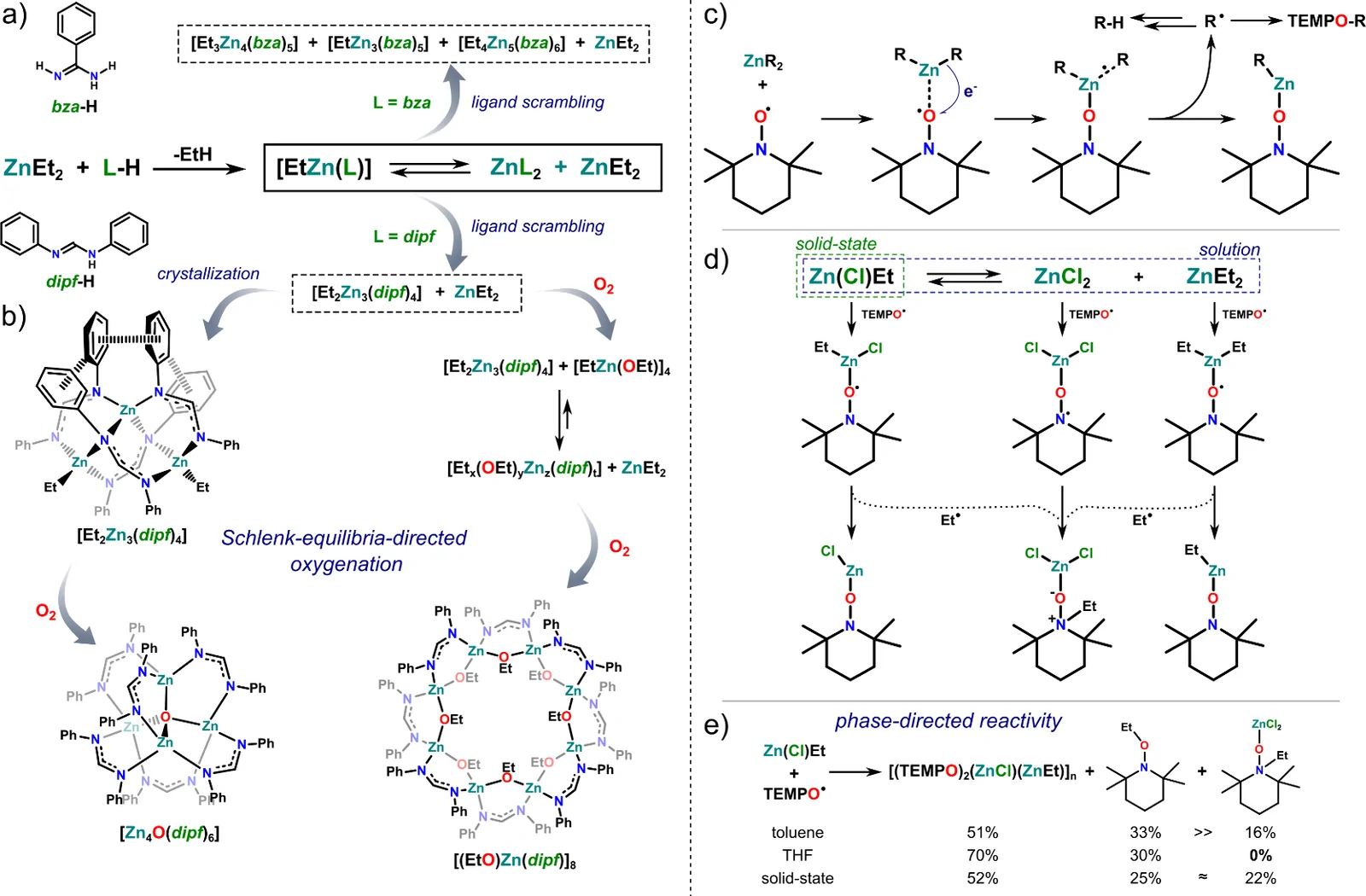
Schlenk equilibria as determinants of organozinc reactivity. (a) Ligand scrambling in alkylzinc amidinates. (b) Speciation-directed oxygenation of the *dipf* system. (c) Stepwise reaction pathway of ZnR_2_ with TEMPO^•^. (d) Reactivity of Zn­(Cl)­Et-derived species with TEMPO^•^. (e) Phase-dependent product distributions for the Zn­(Cl)­Et/TEMPO^•^ system.

Understanding the role of Schlenk equilibria in directing transformations of alkylzinc compounds is essential for the rational development of new reaction pathways. Although complex *bza*-derived reactive ensembles were successfully exploited as precursors for the synthesis of highly stable ZnO nanoplatelets with atomically precise thicknesses in the quantum-size regime,^65,66^ the direct mechanistic role of Schlenk equilibria in controlling reaction outcomes was established through studies on the oxygenation of *dipf* derivatives.^4^ Oxygenation of the *in situ*-generated mixture containing [Et_2_Zn_3_(*dipf*)_4_] and free ZnEt_2_ does not proceed through the dominant heteroleptic aggregate but is mediated by the hidden ZnEt_2_ component, which preferentially reacts with O_2_ to generate [EtZn­(OEt)]_4_ intermediates (Figure 7b). The ethoxide ligands are subsequently transferred to amidinate-containing zinc species through a cascade of ligand redistribution processes, restoring ZnEt_2_ within the reactive ensemble and ultimately leading to the original octameric zinc alkoxide macrocycle [(EtO)­Zn­(*dipf*)]_8_. In contrast, oxygenation of isolated complex [Et_2_Zn_3_(*dipf*)_4_] follows a distinct pathway, yielding the zinc oxo cluster [(μ_4_-O)­Zn_4_(*dipf*)_6_] as the major product.

A complementary demonstration of Schlenk-controlled reactivity emerged from our studies on the reaction of EtZnCl with the TEMPO radical.^2^ In solution, Schlenk redistribution generates a reactive ensemble comprising Et_2_Zn, EtZnCl, and ZnCl_2_, which form simple Lewis acid–base adducts with TEMPO (Figure 7d). Notably, their subsequent reactivity depends strongly on the Lewis acidity of the zinc center. Whereas [Et_2_Zn­(TEMPO)] and [EtZnCl­(TEMPO)] undergo intramolecular electron transfer to generate Et^•^ radicals and predominantly O-alkylated products, the stronger Lewis-acid [ZnCl_2_(TEMPO)] adduct redistributes spin density toward the nitrogen atom, promoting N-alkylation. In turn, in THF solution, solvation of ZnCl_2_ prevents the formation of [ZnCl_2_(TEMPO)] adducts, eliminating the N-alkylation pathway (Figure 7e). In contrast, in the solid state, the polymeric structure of crystalline EtZnCl largely suppresses Schlenk redistribution, localizing reactivity at undercoordinated defect sites that again favor N-alkylation. These results demonstrate how Schlenk equilibria, Lewis acidity, solvent coordination, and phase-dependent speciation cooperate to govern organozinc radical reactivity.

### 2.3 Dynamic Speciation and Reactive Ensembles in Asymmetric Catalysis

Dynamic organozinc speciation is particularly significant in asymmetric catalysis, where identifying the catalytically competent species is essential for understanding and controlling stereochemical outcomes. However, defining the complete reactive ensemble commonly remains challenging because these systems usually consist of multiple rapidly interconverting species. The Soai autocatalytic alkylation of pyrimidine-5-carbaldehydes is a striking example.^15^ Despite more than three decades of study, the species responsible for asymmetric self-amplification remain unresolved, and mechanistic models continue to evolve as new aggregates and intermediates are identified.^16,67,68^ A classical related case is the enantioselective addition of dialkylzincs to aldehydes promoted by chiral amino alcohols. The Noyori model explains the nonlinear effect through an equilibrium between catalytically active, low-coordinate monomeric zinc alkoxides and inactive four-coordinate dimers.^69^ Differences in the stability of homo- and heterochiral aggregates produce the reservoir effect, in which the minor catalyst enantiomer is preferentially sequestered in dormant heterochiral aggregates, thereby amplifying product enantioselectivity (Figure 8b). Although this framework rationalizes many observations, whether the classical monomer–dimer picture adequately captures the underlying speciation remains unresolved.

**Figure 8:**
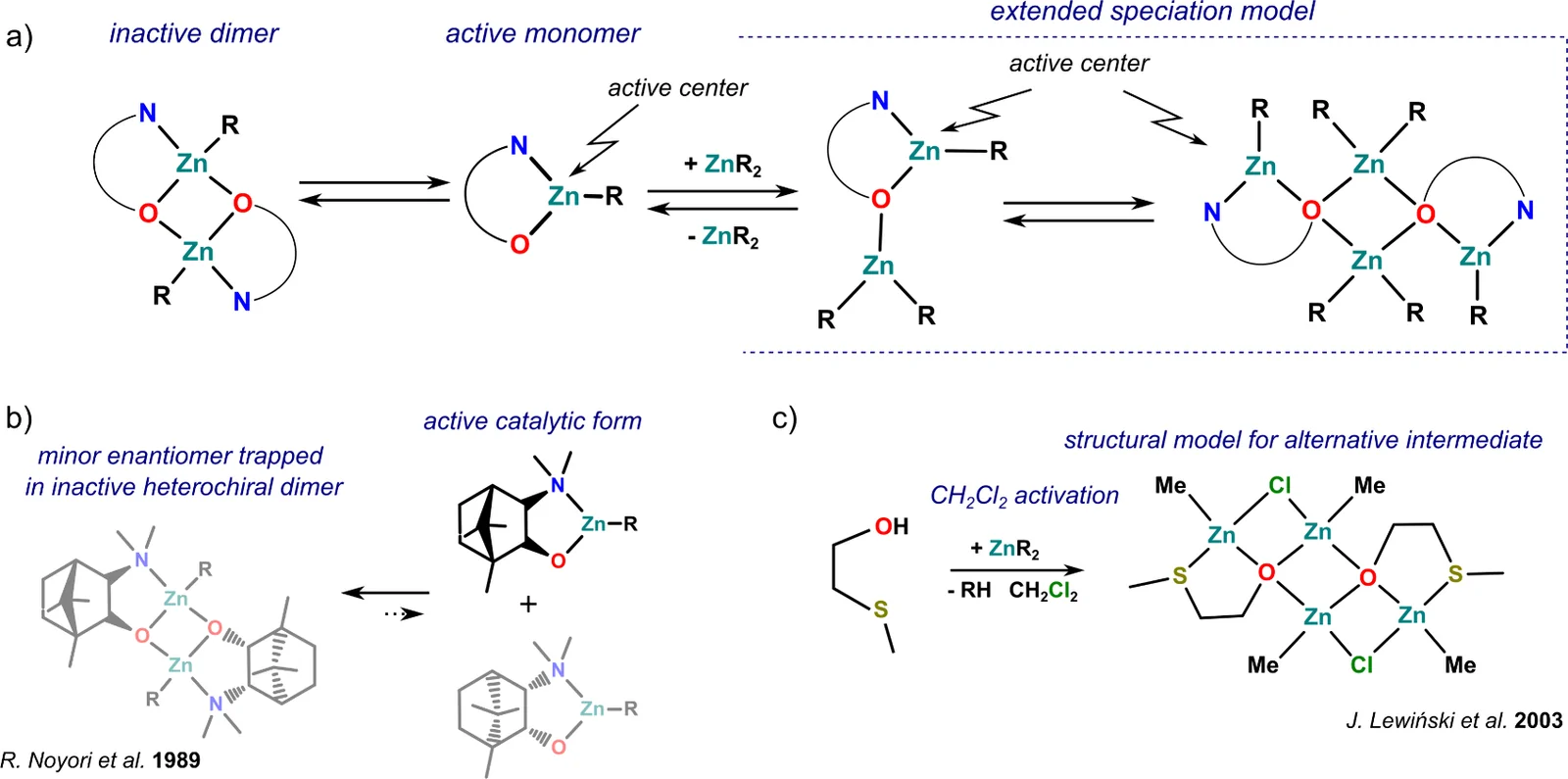
(a) Our alternative speciation model, featuring higher-nuclearity zinc aggregates as catalytically competent species beyond the classical monomer–dimer picture. (b) Classical monomer–dimer equilibria underlying the nonlinear effect. (c) Formation of the tetranuclear zinc aggregate that motivated this alternative interpretation.

In our studies on the reactivity of bifunctional alcohols toward dialkylzincs, we isolated the unprecedented tetranuclear zinc aggregate [MeZn­(OCH_2_CH_2_SMe)­Zn­(Cl)­Me]_2_ from the equimolar reaction of 2-(methylthio)­ethanol with ZnMe_2_ in CH_2_Cl_2_ (Figure 8c).^70^ This structure prompted us to propose an alternative tetranuclear intermediate [RZn­{O,N}­(ZnMe_2_)]_2_ corresponding to the theoretical (ML)_2_ framework invoked by Girard and Kagan to explain nonlinear effects.^71^ Rather than challenging the classical interpretation of asymmetric amplification, our proposal suggests that the reservoir effect extends beyond simple monomer–dimer equilibria (Figure 8a). Catalytically competent low-coordinate zinc centers may originate from higher-order aggregates that preserve the essential features of the (ML)_2_ model while expanding the underlying speciation landscape. Additionally, the unexpected activation of CH_2_Cl_2_ demonstrates that even the reaction medium can actively shape the reactive ensemble, highlighting that solvent effects in organozinc asymmetric catalysis may extend well beyond simple solvation.

A complementary perspective emerges from our studies on the asymmetric epoxidation of α,β-unsaturated ketones catalyzed by zinc alkylperoxides supported by N,N′-bidentate ligands. Although these complexes crystallize as dimers, solution studies established equilibria involving monomeric species as the active catalysts.^72^ Identification of this simple reactive ensemble enabled the rational optimization of catalyst architecture through systematic modification of the ligand framework (Figure 9). Specifically, substituents adjacent to the coordination site decisively influenced enantioselectivity, allowing the most efficient *C*_1_-symmetric enaminooxazoline-derived catalyst to afford epoxides in excellent yields and up to 91% *ee*.^73^

**Figure 9:**
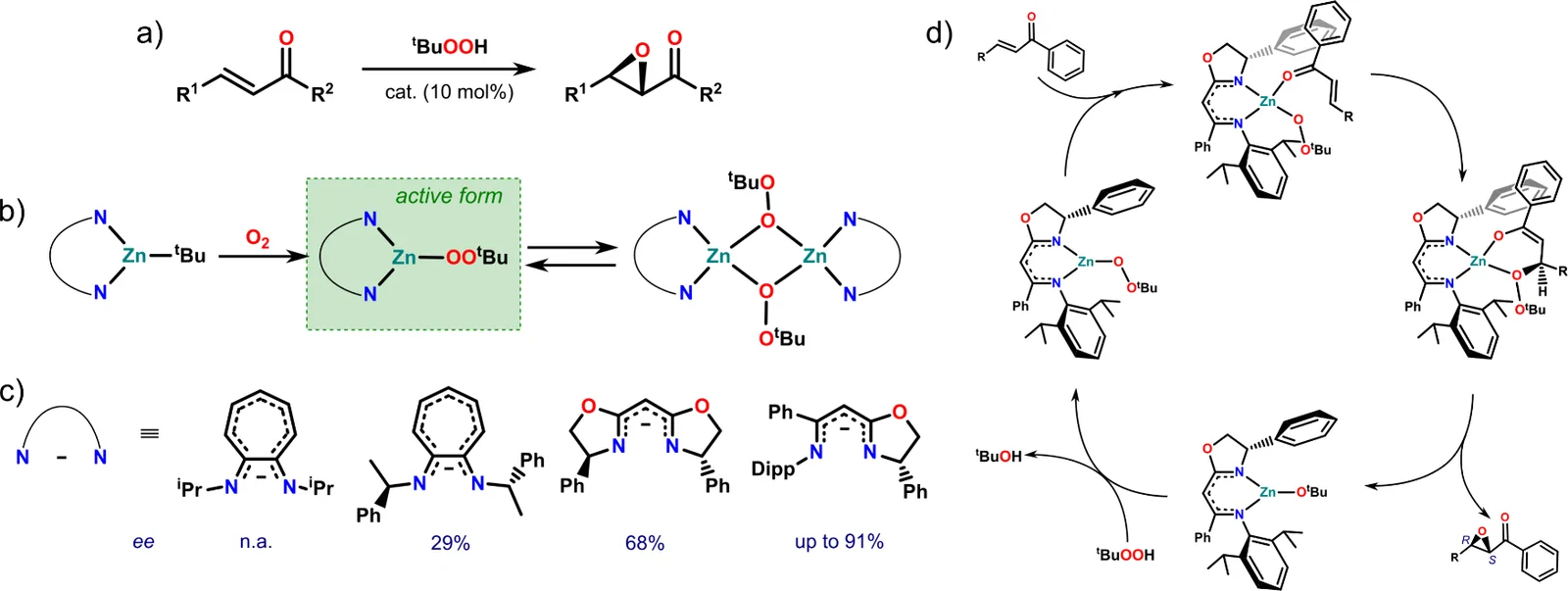
Asymmetric epoxidation of α,β-unsaturated ketones catalyzed by zinc alkylperoxides: (a) Reaction scheme. (b) Catalyst synthesis and speciation. (c) Development of N,N′-bidentate supporting ligands toward improved enantioselectivity. (d) Proposed catalytic cycle.

Overall, these examples demonstrate that the central challenge in asymmetric organozinc catalysis is no longer identifying an individual catalytically active species but understanding how dynamic reactive ensembles generate stereochemical amplification.

### 2.4 Dynamic Speciation in Organozinc Oxygenation: Access to ISET and the Chemistry of Oxygenated Organozinc Species

Since Frankland’s pioneering studies, oxygenation of organozinc compounds has remained one of the most intriguing yet mechanistically controversial transformations in organometallic chemistry.^5^ Long interpreted in terms of free-radical chain mechanisms, it was widely considered intrinsically uncontrollable and of limited synthetic value.^74,75^ We revised this view by showing that organozinc oxygenation proceeds through well-defined, speciation-controlled elementary steps that can be understood and manipulated to achieve selective transformations.^1,25,76^ Because zinc cannot itself act as a redox partner, such systems allow the elementary steps of O_2_ activation to be examined in isolation. The central concept to emerge is the inner-sphere electron-transfer (ISET) mechanism, in which O_2_ approaches a low-coordinate Zn–C center, triggering electron transfer from the Zn–C bond and generating a caged radical pair that collapses to a zinc alkylperoxide (Figure 10a). Consequently, O_2_ activation requires access to a low-coordinate zinc center, making the reaction highly dependent on coordination equilibria. Studies of dialkylzinc Lewis acid–base adducts showed that coordinatively saturated complexes remain essentially inert until ligand dissociation generates reactive three-coordinate species.^25^ This conditionally accessible reactivity defines what we term latent Lewis acidity.

**Figure 10:**
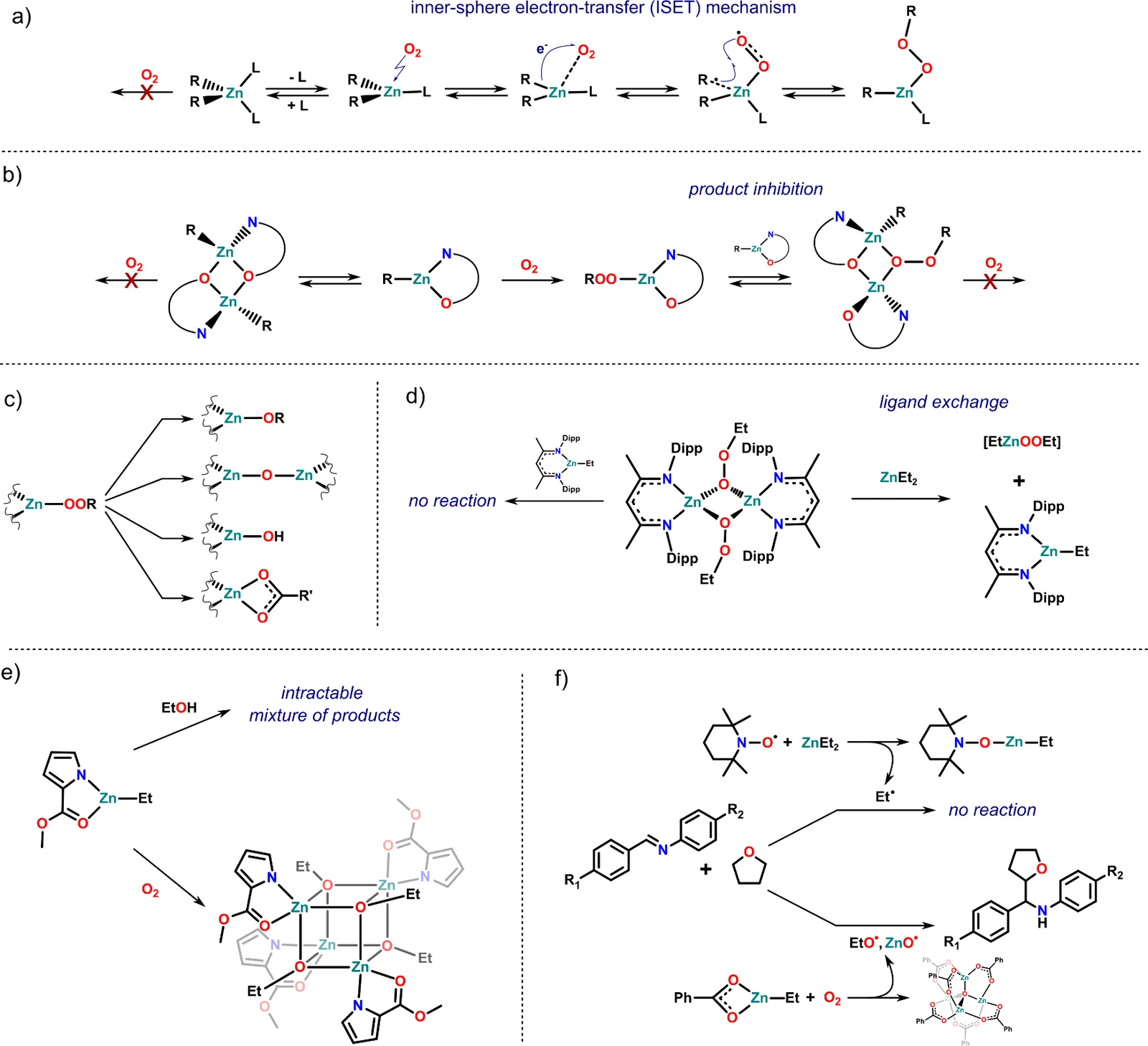
Speciation in alkylzinc oxygenation: (a) ISET mechanism; (b) aggregation equilibria of heteroleptic [RZn­{O,N}] complexes leading to aggregation and product inhibition; (c) diversity of ZnOOR transformation products; (d) ligand redistribution in the [EtOOZn­(BDI)]/ZnEt_2_ system; (e) contrasting outcomes of alcoholysis and oxygenation of the same alkylzinc precursor; (f) oxygen-centered (EtO^•^, ZnO^•^), rather than R^•^, radicals initiate ZnR/air-mediated reactions.

A direct consequence of the ISET mechanism is that oxygenation of ZnR_2_ compounds need not proceed to complete oxidation of both Zn–C bonds but can be terminated at the heteroleptic [RZnOR] alkylzinc alkoxide stage.^1,36^ This process is highly sensitive to coordinating species that modulate access to reactive zinc centers. For example, controlled oxygenation of Zn^t^Bu_2_ in THF at −78 °C selectively affords the alkoxide dimer [^t^BuZn­(O^t^Bu)­(THF)]_2_, whereas the more strongly coordinating 4-methylpyridine suppresses oxygenation at −78 °C and, at higher temperature, stabilizes the corresponding alkylperoxide [^t^BuZn­(OO^t^Bu)­(py-Me)]_2_.^25^ For less sterically demanding systems, the initially formed RZnOR species assemble into coordinatively saturated [RZn­(OR)]_4_ heterocubanes, which suppresses further oxygenation by removing oxygenation-competent low-coordinate zinc centers. This change in coordination environment provides a mechanistic explanation for the frequently noted apparent inertness of the second Zn–C bond during ZnR_2_ oxygenation. The second bond is not intrinsically less reactive; rather, the introduction of OOR/OR ligands drives bridging and coordination saturation (commonly forming four-coordinate zinc centers). Because O_2_ activation requires access to a low-coordinate, Lewis-acidic site, this product-modified reactive ensemble kinetically blocks further oxygenation.

The isolation of the first structurally authenticated zinc alkylperoxides enabled direct investigation of their chemistry and revealed how oxygenated products can reshape reactive ensembles. Oxygenation of the three-coordinate β-diketiminate (BDI) complex [EtZn­(BDI)] afforded the dimeric zinc ethylperoxide [(EtOO)­Zn­(BDI)]_2_, establishing Zn–OOR species as isolable organometallic compounds.^76^ In turn, aminoalcoholate-supported ethylzinc complexes yielded the mixed aggregate [EtZn­{O,N}]_2_[(EtOO)­Zn­{O,N}]_2_, where alkylperoxide and parent alkylzinc units coexist (Figure 10b).^1^ This structure provided direct evidence for the product inhibition effect: the alkylperoxide product associates with the parent alkylzinc species to form dormant aggregates, rendering oxygenation self-limiting after partial conversion of Zn–C bonds. A related principle was later observed in ZnR_2_/diketone systems, where alkylated zinc alkoxides form dormant aggregates with ZnR_2_ and inhibit further alkyl transfer.^77^ Thus, products can act as active components of reactive ensembles rather than passive endpoints.

**Figure 11:**
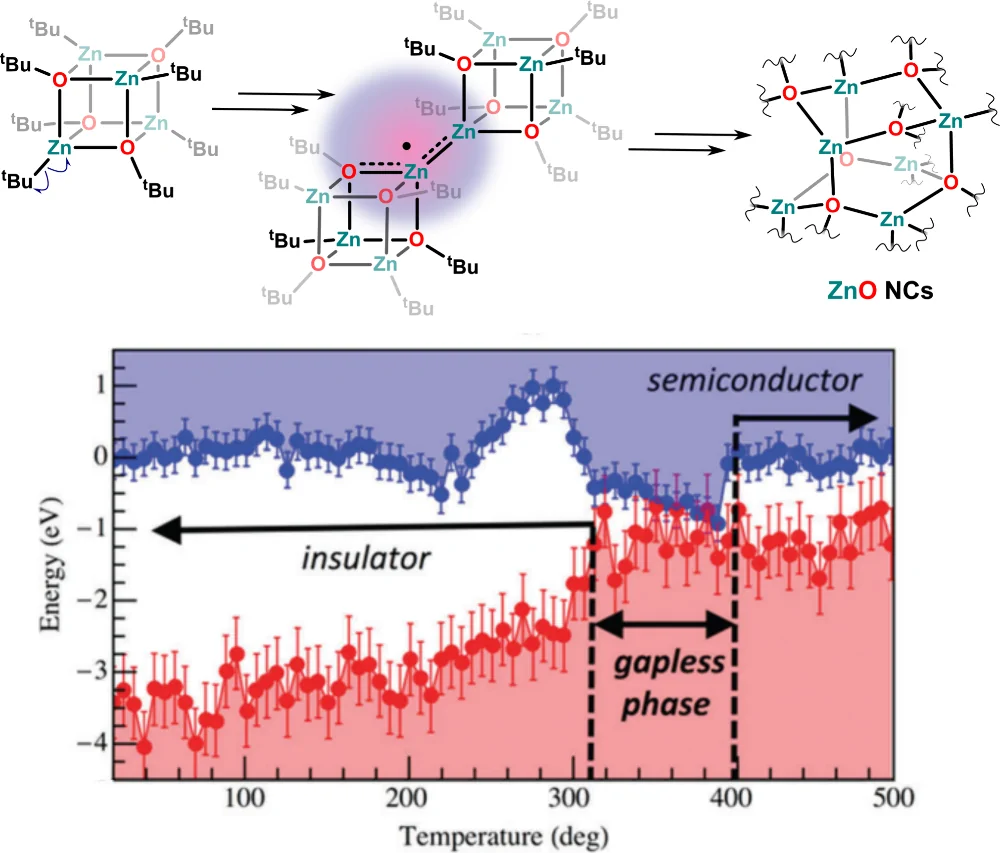
The thermal transformation of preorganized zinc alkoxides into ZnO nanocrystals, including the emergence of transient gapless states, was elucidated using advanced core-level spectroscopic techniques combined with theoretical calculations. Adapted with permission from ref 31. Copyright 2018 Royal Society of Chemistry.

Subsequently, a key mechanistic assumption that could be tested experimentally was the proposed peroxide–alkyl metathesis between Zn–OOR and Zn–R species, invoked to explain the formation of zinc alkoxides during the oxygenation.^75^ Contrary to this classical view, reaction of [(EtOO)­Zn­(BDI)]_2_ with parent [EtZn­(BDI)] did not result in peroxide–alkyl metathesis and in the case of ZnEt_2_ instead proceeded through ligand redistribution, affording [EtZn­(OOEt)] and regenerating [EtZn­(BDI)] complex (Figure 10d).^78^ This finding challenged a central element of the classical oxygenation model and established ligand redistribution as a genuine pathway in oxygenated organozinc chemistry. Further studies showed that Zn–OOR species undergo system-dependent transformations, leading to diverse oxygenated products, including zinc alkoxides,^64,78–80^ hydroxides,^78^ carboxylates,^78,81^ and oxo clusters (Figure 10c).^4,46,82^ We identified homolysis of the ZnO–OR unit as the dominant pathway, a route largely overlooked, since homolytic O–O cleavage is typically associated with redox-active metals. These findings revealed that oxygen-centered radical pathways can be selectively controlled within well-defined organozinc frameworks rather than representing uncontrolled radical-chain processes. The synthetic value of this understanding was demonstrated, inter alia, by the controlled oxygenation of [RZn­{O,N}]-type complexes supported by 2-ester-substituted pyrrolate ligands, which selectively afforded well-defined zinc alkoxides, including trimeric [(^t^BuO)­Zn­{O,N}]_3_ and heterocubane-type [(EtO)­Zn­{O,N}]_4_.^83^ Notably, these alkoxides were inaccessible by conventional alcoholysis, which instead produced ill-defined or structurally distinct products, highlighting oxygenation as a complementary route to zinc alkoxide complexes relevant to catalytic applications (Figure 10e).

Understanding these mechanistic pictures prompted a fundamental reassessment of ZnR/air-initiated radical organic reactions. The prevailing model, reiterated in Tomioka’s Account, assigned initiation to R^•^ species.^17^ Our comparative studies instead identified O-centered radicals as the true initiators and translated this insight into the rational development of efficient RZn­(L)/air initiation systems for organic synthesis (Figure 10f).^18^ This work, therefore, not only revised the mechanistic foundation of the field but also established a design framework for controlling radical generation through organozinc speciation and O_2_ activation. More broadly, organozinc oxygenation emerges not as an intrinsically uncontrolled radical process, but as a dynamic reaction network in which access to ISET, ZnOOR reactivity, ligand redistribution, product inhibition, and radical identity are governed by the evolving reactive ensemble. This ensemble picture introduces a further mechanistic dimension. Dynamic speciation may govern not only which Zn–C unit encounters O_2_, but also the electronic properties of O_2_ during this encounter. Our recent calculations indicate that the local electrostatic environment around redox-silent zinc centers perturbs the O_2_ π* acceptor manifold before charge transfer. Thus, the reactive ensemble controls both the availability of the electron source and the readiness of O_2_ to accept it.^84^

## 3 Phase-Dependent Speciation and Solid-State Reactivity Modulation

Given the strong dependence of organozinc speciation on its chemical environment, the reactive ensemble may differ substantially between solution and the solid state, making phase an additional variable governing reactivity. As illustrated above by the EtZnCl/TEMPO system, phase-dependent speciation can profoundly alter reaction pathways, while the structural evolution of [^t^BuZn­(O^t^Bu)]_x_ aggregates shows that the solid state can stabilize otherwise inaccessible organozinc assemblies (vide supra). Accordingly, dynamic speciation is not limited to solution chemistry but may also play a decisive role in governing solid-state transformations. For example, [RZn­(OR)]_4_ heterocubanes have long been used as single-source precursors for ZnO nanocrystals, yet their thermolysis does not represent a simple conversion of a molecular precursor into a semiconductor. Our in situ v^2^c-XES/HEROS studies of [^t^BuZn­(O^t^Bu)]_4_ revealed transient open-shell zinc oxo/alkoxide clusters with Zn-centered radical character and emerging Zn–Zn interactions, defining an electronic speciation regime between molecular organozinc compounds and ZnO nanocrystals (Figure 11).^31^ Thus, precursor conversion involves not only structural reorganization but also the emergence of new electronic states.

This solid-state perspective was further extended to supramolecular modulation of organozinc reactivity. Alkylzinc systems incorporating the diphenylmethanol, or “magic carbinol”, unit exploit geminal phenyl groups to stabilize unusual aggregation states through C–H···π and Zn···π interactions (Figure 12a). This motif ultimately enabled supramolecular encapsulation of pyrophoric ZnR_2_ reagents within crystalline sponge hosts.^3^ The host frameworks stabilize otherwise highly reactive dialkylzinc liquids, allow crystallographic characterization of confined ZnR_2_ molecules, and enable selective separation of ZnMe_2_ from ZnMe_2_/ZnEt_2_ mixtures. More importantly, confinement reshapes the reactive ensemble by controlling aggregation, donor accessibility, and O_2_ exposure. In this sense, the crystalline sponge functions as a higher-order ligand: it governs organozinc speciation through the supramolecular environment rather than through direct coordination to zinc.

**Figure 12:**
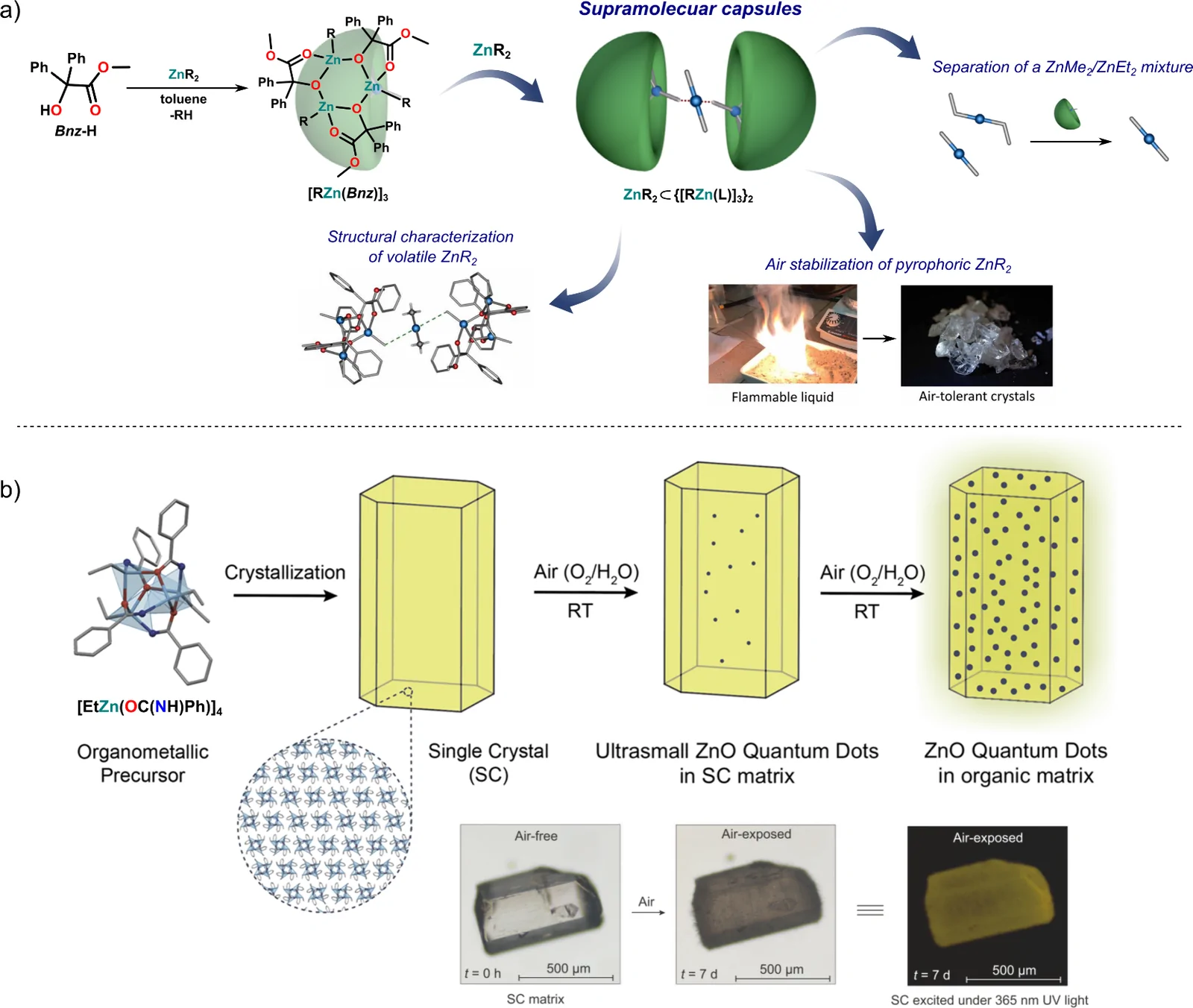
Modulation of solid-state reactivity of alkylzinc compounds. (a) Supramolecular encapsulation of ZnR_2_ species. Adapted from ref 3. Available under a CC-BY 4.0 license. Copyright 2025 K. Sokołowski et al. (b) Solid-state transformations of ethylzinc benzamidate single crystals toward monolithic ZnO QDs assemblies. Adapted from ref 85. Available under a CC-BY 4.0 license. Copyright 2025 A. Borkenhagen et al.

The culmination of this solid-state and supramolecular trajectory was the spontaneous chemical weathering of organozinc molecular single crystals into monolithic assemblies of ZnO quantum dots under ambient conditions (Figure 12b).^85^ In this transformation, water is not merely an atmospheric trigger but the key chemical reagent. Atmospheric moisture penetrates the initially nonporous [EtZn­{OC­(NH)­Ph}]_4_ crystal, where it hydrolyses the Zn–C and Zn–N bonds, generating Zn–OH/Zn–O species and regenerating benzamide. Remarkably, although the crystallographic order of the molecular precursor is rapidly lost, the macroscopic shape and volume of the parent single crystal are retained. The hydrolyzed ligands and absorbed water create an evolving hydrogen-bonded matrix that acts as a confined, solvent-like reaction medium, enabling mass transport and spatially directing prenucleation, nucleation, and growth of ca. 4.5 nm ZnO quantum dots. Removal of this organic matrix ultimately leaves a mesoporous monolithic scaffold composed of quantum-sized ZnO domains. Thus, the parent crystal does not transmit its lattice structure directly to the product; rather, its confined molecular architecture is converted during reaction into a dynamic speciation environment that controls where and how the inorganic nanophase emerges. In this sense, solid-state organozinc chemistry becomes a route in which molecular precursor design, reaction-induced speciation, and supramolecular confinement collectively direct the transformation from molecular crystals to functional inorganic materials.

## 4 Conclusions and Outlook

The central message of this Account is that organozinc reactivity is governed not by isolated molecular structures but by dynamic speciation landscapes comprising interconverting aggregates, donor adducts, Schlenk-derived species, reaction intermediates, radical pairs, and phase-confined assemblies. Reaction outcomes therefore emerge from the composition and evolution of these reactive ensembles rather than from any single molecular species. This perspective reframes organozinc chemistry: oxygenation can be understood as a sequence of speciation-controlled transformations rather than an intrinsically uncontrolled radical process, while Schlenk equilibria, aggregation, donor coordination, and phase or supramolecular confinement emerge as active determinants of reaction pathways.

Ultimately, the reactive species is often a transient member of an evolving ensemble. A key challenge is therefore to make these ensembles observable and controllable by combining in situ spectroscopy, diffraction, computation, and kinetic analysis under operating conditions. Equally important is treating aggregation, donor coordination, Schlenk equilibria, and confinement as design variables that define both species distribution and the local environment experienced by incoming reagents. Linking such ensemble-controlled environments to elementary electron-transfer energetics represents an important frontier for organozinc chemistry.

## ∇

I.J. passed away on July 29, 2026.
